# Supplementary material for: Predicting visual function by interpreting a neuronal wiring diagram
Source: Nature. 2024 Oct 2;634(8032):113–23. doi: 10.1038/s41586-024-07953-5 (PMC11446822; doi:10.1038/s41586-024-07953-5)
Supplement: Supplementary file 6 — CRF and ERF predictions for individual TmY4 and TmY9 cells. Analogous to Supplementary Data 3, but for TmY target types. Shown are the top four monosynaptic pathways, the strongest pathway passing through each of the top ten intermediary types (ranking from Extended Data Fig. 7), and the trisynaptic pathway Tm1–TmY–Dm3–TmY (see the section entitled Prediction of spatial normalization). [file 41586_2024_7953_MOESM6_ESM.zip › DataS4/TmY9q/720575940624293417.pdf]

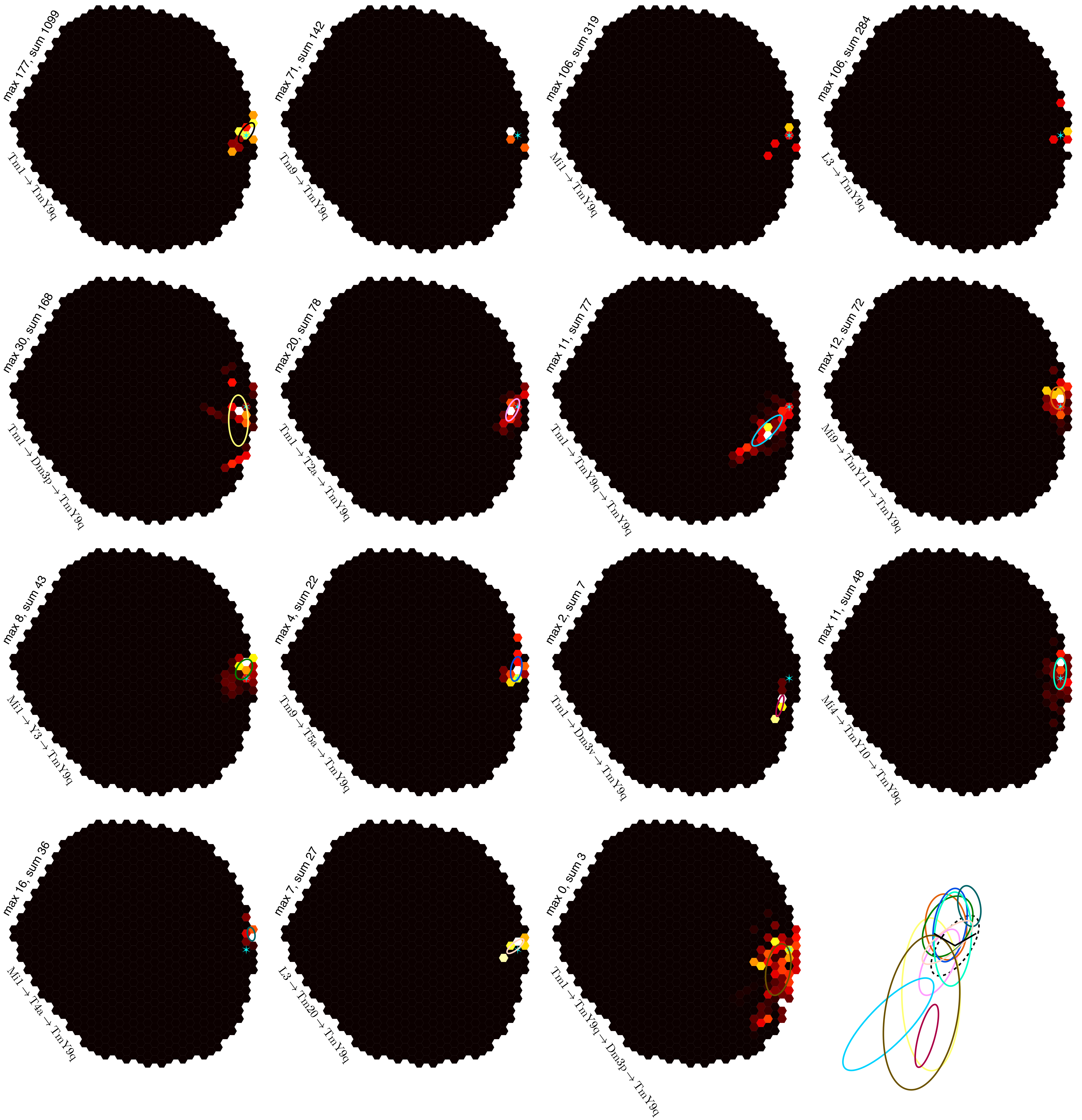

max 177, sum 1099
Tm1 → TmY9q
max 71, sum 142
Tm9 → TmY9q
max 106, sum 319
Mi1 → TmY9q
max 106, sum 284
L3 → TmY9q
max 30, sum 168
Tm1 → Dm3p → TmY9q
max 20, sum 78
Tm1 → T2a → TmY9q
max 11, sum 77
Tm1 → TmY9q → TmY9q
max 12, sum 72
Mi9 → TmY11 → TmY9q
max 8, sum 43
Mi1 → Y3 → TmY9q
max 4, sum 22
Tm9 → T5a → TmY9q
max 2, sum 7
Tm1 → Dm3v → TmY9q
max 11, sum 48
Mi4 → TmY10 → TmY9q
max 16, sum 36
Mi1 → T4a → TmY9q
max 7, sum 27
L3 → Tm20 → TmY9q
max 0, sum 3
Tm1 → TmY9q → Dm3p → TmY9q
